# Micro Injection Molding of Thin Cavities Using Stereolithography for Mold Fabrication

**DOI:** 10.3390/polym13111848

**Published:** 2021-06-02

**Authors:** Rossella Surace, Vito Basile, Vincenzo Bellantone, Francesco Modica, Irene Fassi

**Affiliations:** 1 Institute of Intelligent Industrial Technologies and Systems for Advanced Manufacturing, National Research Council (STIIMA-CNR), 70124 Bari, Italy; vito.basile@stiima.cnr.it (V.B.); vincenzo.bellantone@stiima.cnr.it (V.B.); francesco.modica@stiima.cnr.it (F.M.); 2 Institute of Intelligent Industrial Technologies and Systems for Advanced Manufacturing, National Research Council (STIIMA-CNR), 20133 Milano, Italy; irene.fassi@stiima.cnr.it

**Keywords:** micro injection molding, mold, thin cavity, additive manufacturing, stereolithography

## Abstract

At the present time, there is a growing interest in additive manufacturing (AM) technologies and their integration into current process chains. In particular, the implementation of AM for tool production in micro injection molding (µ-IM), a well-established process, could introduce many advantages. First of all, AM could avoid the need for the time-consuming and expensive fabrication of molds for small series of customized products. In this work, the feasibility, quality, and reliability of an AM/µ-IM process chain were evaluated by designing and fabricating mold inserts for µ-IM by stereolithography (SLA) technology; the mold inserts were characterized and tested experimentally. The selected geometry is composed of four thin cavities: This particular feature represents an actual challenge for both the SLA and µ-IM perspective due to the large surface-to-volume ratio of the cavity. Two different materials were used for the mold fabrication, showing sharply different performance in terms of endurance limit and cavity degradation. The obtained results confirm that the µ-IM process, exploiting an SLA fabricated mold insert, is feasible but requires great accuracy in material choice, mold design, fabrication, and assembly.

## 1. Introduction

Injection molding of micro or thin components is a widespread technology due to its capability of manufacturing low-cost and highly repeatable polymeric parts relevant to many different fields, from IT to healthcare to the biomedical sector. However, the high costs of mold design and manufacturing via conventional approaches based, i.e., on milling/EDM processes affect the development cycle and industrial uptake of new products. AM technologies offer several advantages with respect to traditional manufacturing for mold and insert fabrication, such as waste reduction, minimized energy consumption, reduced overall manufacturing time, and time to market. The company can adapt or adjust the new product design step by step according to the requirements assessed by the end users so that when a stable design is reached, the production can be scaled up and transferred to a mass production process, thus reducing the investment risk and the time to market. Furthermore, even if only a small series or single-unit batch are required, the additive manufactured mold could cause the injection process to be economically feasible [1,2]. The last relevant advantage of using these technologies for mold production, especially for traditional molding, relies on the possibility to easily design and fabricate molds with a conformal cooling system, thus not only reducing cooling time and cycle time, but also increasing the product quality by optimizing shrinkage and reducing warpage [3].

Among additive technologies for polymers, stereolithography is the process that best fits the compromise between quality (accuracy and surface finishing), cost, and ease of use. The “soft” materials, such as polymers, used in AM processes for mold fabrication present thermal and mechanical properties and toughness that are significantly different from those of steel, affecting both the tool durability at high temperature and the quality of the final products [4]. In particular, the tool failures occur during the injection phase due to the flow pressure in connection with high thermal stress and during the extraction phase due to the increasing stress [5,6]. 

At a macro scale, the use of additive manufactured molds has been studied by different authors and the achieved results are different in terms of duration and quality of the parts due to the different applied additive technologies and materials. In terms of quality, comparing three mold prototypes for injection molding realized by different AM technologies (stereolithography—SLA, selective laser sintering—SLS, and resin photo-polymerization—3D Polyjet), Leon-Cabezas et al. [7] found out that the best mold was the one fabricated by photo-polymerization. Indeed, the SLA mold showed curved surfaces after curing, whereas the SLS mold suffered from low surface quality. In terms of mold duration, the literature reports that an epoxy SLA mold was able to produce more than 500 plastic parts with acceptable quality [4], whereas a fused deposition modeling (FDM) mold realized about 50 shots, showing comparable quality with parts obtained using a steel mold [8]. 

More recently, new materials have been developed for several AM technologies, relaunching research on this topic. Dizon et al. [9] presented a wide and exhaustive prospective article on injection molding and 3D-printed molds and materials, showing two relevant test cases: IM using SLA and FDM 3D-printed mold inserts. In both cases, performance, accuracy, and mold insert failure mechanisms were presented. SLA was more accurate and gave higher surface finishing than FDM. The weakness of the SLA molds was referred to as cracking, and chipping mechanisms occurred after 60 shots, which were mainly attributed to transverse tension and the thermal cycle, whereas layer delamination strongly limited (10 shots) the endurance of the FDM 3D-printed mold inserts. Again, the same authors [10] conducted injection molding tests with SLA, polyjet, and FDM mold inserts. A PEEK mold fabricated by FDM showed delamination, whereas mold inserts made by SLA and polyjet technologies showed good surface finishing and acceptable dimensional accuracy and stability, below 5%.

An experimental material characterization for mold insert applications was proposed by Etesami et al. [11] by measuring bending deflection and surface hardness of material specimens at high temperatures. Four materials for different AM technologies were compared; the results showed that composite materials, such as Markforged fiberglass-filled HSHT filament, exhibit lower deflections and higher surface hardness at high temperatures up to 170 °C. Good performance was evidenced by Formlabs High Temperature SLA resin, whereas poor properties were measured by ABS and ONYX filaments for FDM. Mechanical properties and tensile and compression limits of some Formlabs SLA resins have been also experimentally verified for mold insert fabrication [12], showing that the limits can be much lower than those declared by manufacturers in material datasheets. In addition, regarding material performance, the authors in [13] demonstrated that the design rules of mold inserts and mold assembly setup [14] are very important to success, avoiding defects and allowing for a high number of shots before mold failure. 

Considering the micro world, which this paper is focused on, some experimental works were already done in recent years, especially by Tosello et al., about the fabrication of bricks (dimensions 5 × 4 mm) and cylindrical pillars (height and diameter 200 µm) molded by soft tools carried out via digital light processing (DLP) technology. In particular, these authors focused on thermal numerical modeling for tool life evaluation [15], cost analysis, and the optimization [16,17] and quality of molded parts [18,19,20]. In terms of the quality and morphology of molded parts, it is possible to increase crystallinity using a jetted photopolymer mold that reduces the rate of cooling due to the low thermal conductivity of the used digital ABS [21]. In reference [22], DLP inverted stereolithography was used to fabricate mold inserts with microfeatures consisting of an array of 100 μm-wide positive micro-channels with heights and aspect ratios in the ranges of 24–94 μm and 0.2–0.7, respectively. The results show that DLP had similar performance as SLA in terms of accuracy and surface finishing of realized parts. In addition, cracking and chipping failure mechanisms were the same. Vasco et al. [23] compared selective laser melting (SLM) and high-precision SLA to fabricate metal and resin mold inserts, respectively. These technologies were tested with various geometries made with pillars 100 μm wide and 200 μm high. SLA guaranteed high surface finishing, whereas SLM resulted in poor surface quality that could be improved by electron-beam polishing. SLA is more suitable for micro-featured mold inserts and μ-IM but only for short runs, due to cracking and damage to cavities caused by the injection flow. Conversely, SLM metal mold inserts have thermal and mechanical properties similar to traditional steel mold inserts and therefore are quite suitable for long runs. Finally, Gheisari et al. [24] studied SLA resins and found out that they must have high a glass transition temperature and low thermal conductivity to be suitable for mold making. Table 1 reports the performance of AM mold inserts in micro-injection molding.

In this work, focusing on the field of micro-manufacturing, the combination of stereolithography mold manufacturing and the micro-injection process is proposed to take advantage of both techniques. A mold with four thin cavities with a large surface-to-volume ratio was chosen as the test case since it represents a challenge for both processes, SLA and µ-IM. The mold insert for the µ-IM process was designed following mold-insert design rules and the specifications of AM production, and then carried out via SLA with two different materials that are described later: grey resin (G) and high-temperature resin (HT). The two molds were characterized to evaluate the achieved dimensional and geometrical precisions of the stereolithography process and experimentally tested in the micro-injection molding machine to evaluate the replication capability and durability in terms of number of shots to failure (NSF), and thus the feasibility, accuracy, and reliability of the proposed SLA/µ-IM process chain. 

## 2. Mold Insert Design 

**Figure 1:**
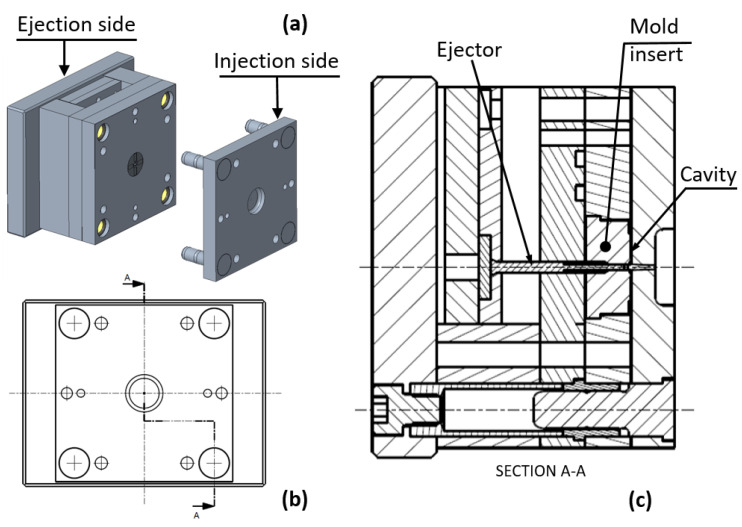
Mold assembly: (**a**) 3D model of the mold, (**b**) side view with section A-A trace, and (**c**) drawing of the mold at section A-A.

The proposed mold is composed of two main parts: the mold base, which is the main assembly structure equipped with all subsystems (cooling, ejection, injection, cold/hot distribution, etc.), and the mold insert with shaped cavities, injection feeding, and melt distribution (cold runners). Typically, both the mold base components and mold insert are made of steel and, as mentioned, their manufacture requires relevant design and machining time. In this work, the mold insert was fabricated by additive manufacturing, thus significantly shortening its fabrication time. Figure 1 shows the 3D model of the mold (a) with main sub-assemblies labeled and a section drawing (b–c) where the mold insert is highlighted. 

**Figure 2:**
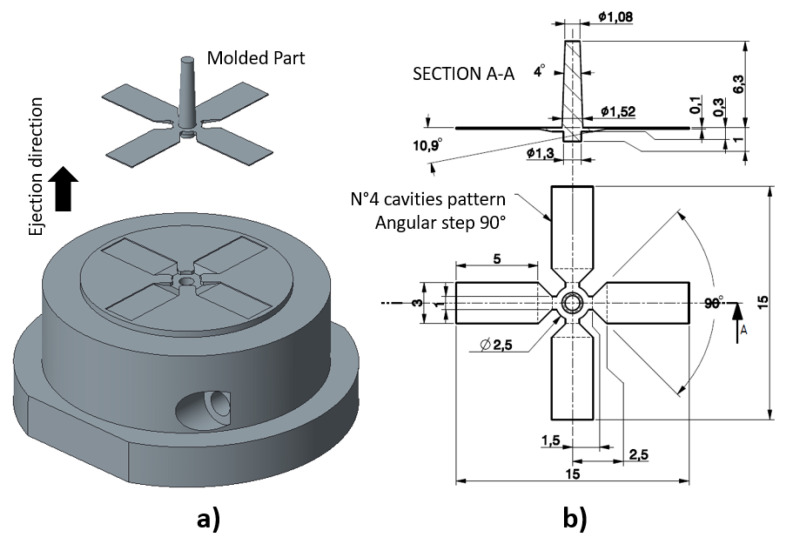
Mold insert with notation of ejection direction (**a**) and molded part drawing (**b**).

**Figure 3:**
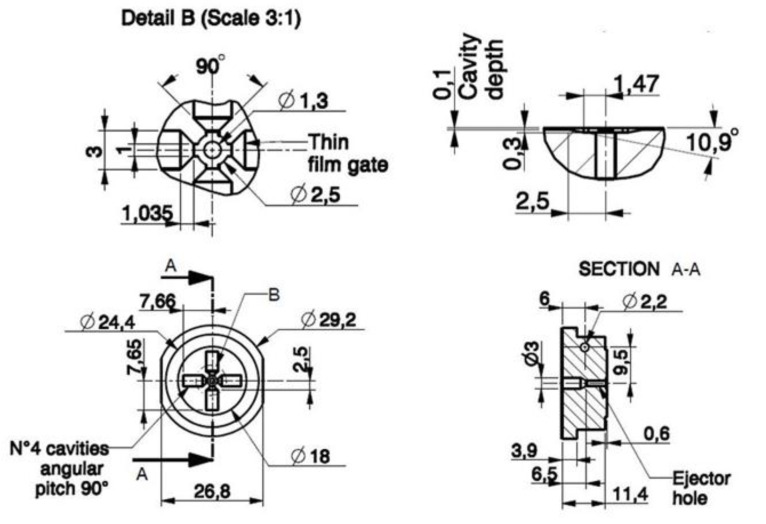
AM mold insert drawing with cavity dimensions.

The mold insert design is an important task in the development process since it should address issues of both fabrication (AM for mold insert fabrication) and final processing (μ-IM) technologies. The first step is the part assessment, which includes material selection, geometric features (especially considering the very thin thickness of the part), and part design guidelines [25]. When the part design is approved, then the mold insert design can be performed by addressing several critical aspects, such as moldability, parting surfaces, number and layout of cavities, draft angle of walls normal to the extraction direction, distribution channels, gates, molded part retaining system before ejection, ejection, cavity vents, and material shrinkage compensation. Figure 2 and Figure 3 show the mold insert designed for AM fabrication, the detailed and 3D drawing views, and the part nominal dimensions. Once mounted on the injection machine, the mold insert is instrumented with a thermocouple probe, thus monitoring its temperature during the molding process (hole in Figure 2a). 

The designed mold insert presents four cavities radially distributed with a nominal depth of 100 μm, thin film gates, and one central cylindrical ejector pin with a nominal diameter of 1.3 mm, guided for a length of 5 mm. Filling these cavities without defects is already a challenge for the common micro-injection molding that uses a steel mold. The challenge is even greater for the combination of injection molding using an SLA mold, because of the mechanical and thermal properties of the resin mold and the need for relatively high temperature and pressure to fill the cavities. Limitations can also arise from the interaction between the resin insert and melt polymer. However, the design has the goal of defining a sort of metric by measuring the infill length to judge this unusual application. 

In this work, four challenging aspects arose: (i) filling of the thin cavities, (ii) part quality and accuracy, (iii) ejection of the part, and (iv) mold insert design for durability. In order to address the first issue (cavity filling), thin film gates were adopted to guarantee a full-width and uniform feeding of the melt polymer into the thin cavities. To this scope, starting from the central injection sprue with a diameter of 2.5 mm, the melt front was split into 4 gates 1 mm wide and 0.3 mm deep, which arrived at the cavities with gates 3 mm wide and 0.1 mm deep, therefore having a trapezoidal design with a distance between the two gates of about 1 mm. It is worth noting that the design of the gates must fulfil the rule of preserving the cross sections of the gates. In this design it was set to 0.3 mm^2^. The two gates are very close (1 mm), therefore the melt injection pressure will not differ between them if the cross sections are invariant. This design rule for thin film gates allows a uniform melt flow to the cavity without pressure drops, thus succeeding in filling. 

With regards to the second issue (part quality and accuracy), the main concern is represented by the selection of optimized 3D-printing parameters in order to achieve high surface finishing and dimensional/geometrical accuracy. 

The third issue, the ejection of the part, is a critical aspect of the design since it can also cause the occurrence of failures. To address this issue, the designer must consider the mechanical stress arising on the mold insert during the injection molding and the required force to overcome the adhesion between molded parts and cavities. Leveraging the very low part thickness, in this work, the ejection was performed by a cylindrical pin with a diameter of 1.3 mm placed on the sprue at the center of the mold insert, thus pushing on a thicker region of the molded part and enabling a radial peeling of the parts from the cavities. This design rule also allows the overall molded part to be retained at the ejection side of the mold, preserving the ejection effectiveness. The fit between the pin ejector and the hole should be tight and the guidance into the mold holes should be high in order to not induce stress on the mold and avoid the occurrence of flashings. A maximum value of clearance of 30 μm is assumed as the limit for the referred tolerance. In addition, the wearing caused by the sliding steel ejectors can be very high since SLA material hardness is low. The main rule to improve the sliding of ejectors and avoid SLA mold breakages is the reduction of ejection force, which can be achieved by tapering the overall mold insert cavity. Since the molded part has very low thickness (100 μm), the cavity surfaces do not contribute to increasing the extraction force and can be neglected. On the contrary, the sprue and gate surfaces should have a draft angle along the extraction direction, typically higher than 1 degree. Since the ejection force is inversely proportional to the cavity surface roughness, another design rule to fulfill is to have high surface finishing of cavities. In this test case, ejector pins were not present in the cavities in order to not compromise the quality and the moldability of the parts. In fact, considering the very low thickness of the part, ejectors in the cavity would require high accuracy in assembly, and the ejection force and thrust could rip the part and, in better cases, mark the molded parts with the pin profile.

The last issue (design for durability) should be addressed by analyzing the failure mechanisms affecting the AM mold inserts and particularly the 3D-printed SLA. As known by the state of the art summarized in Table 1, chipping and cracking failures occur on SLA mold inserts. Cracking is mainly caused by transverse tension and thermal cycle [9], but stress concentrations on the edges can also trigger the cracks. In the proposed test case, the higher risk of cracking and chipping was limited to the center of the mold insert where deep edges were present. 

## 3. Mold Insert Fabrication Via SLA

Stereolithography (SLA) is a well-known technology capable of printing parts with high dimensional accuracy, smooth surface finishing, and high feature resolution, made with polymers with good mechanical properties. These features make this technology particularly suitable for small parts fabrication with challenging 3D shapes and geometry features with high surface quality. 

**Figure 4:**
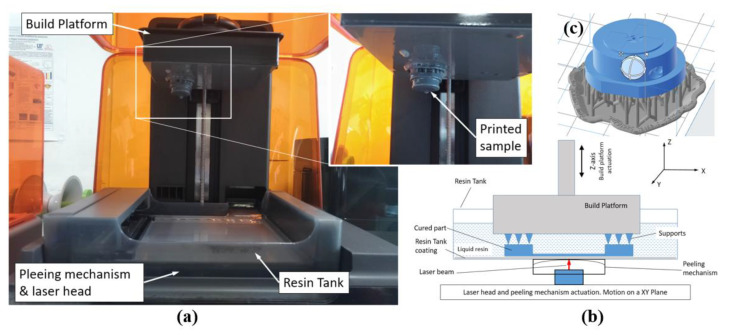
Mold insert manufacturing by SLA: (**a**) bottom-up SLA Formlabs Form3 machine at the end of the 3D-printing process, (**b**) conceptual scheme of the inverted SLA technology, and (**c**) part pre-processing with slicer software Formlabs Preform.

In this work, the SLA 3D printing of the micro-mold insert was performed by means of a Form3 machine (Figure 4a) (Form3, Formlabs, Somerville, MA, USA), which implements bottom-up exposure (inverted) stereolithography [24]. In this version of the process, once the current layer is completely processed, a peeling mechanism performs the detachment of the polymerized layer from the resin tank [26]. If not accurately controlled, the peeling phase can be critical for the geometrical accuracy of the part [27]. This 3D printer is characterized by a build volume of 145 × 145 × 185 mm^3^ and a light source comprising a class 1 violet laser emitting at a wavelength of 405 nm with a power of 250 mW and a spot diameter of 85 μm. The 3D printer allows a positioning resolution of 25 μm on the XY plane (layer) to be achieved, and the resolution along the *z*-axis can be set to 200, 100, 50, or 25 μm (layer thickness). 

The material properties of the mold insert must also be considered: thermal conductivity, melting and softening temperatures, mechanical strength (yield and ultimate stress limits), stiffness in the elastic range (Young’s modulus), hardness, and impact sensitivity (IZOD). In the past, thermal and mechanical properties of AM materials significantly limited the quality and endurance life of the AM molds, but recently, new materials with improved performance have been developed for these applications. In order to investigate material properties influence, the mold inserts were fabricated using two Formlabs resins: Standard Grey Resin V04 (G) and High Temperature V02 Resin (HT). The mechanical and thermal properties of these two materials are reported in Table 2 [28]. This table gives the values for three different cases: no post-processing (green part), only UV curing, and UV curing with additional annealing at a high temperature. Post-processing (UV curing and annealing) increases almost all mechanical properties of the materials. The heat deflection temperature (HDT) was sharply increased by UV curing with variations in the range of 37–145%, and by an additional increment of 241% by thermal treatment (only HT resin). The final values of HDT at 0.45 MPa of the two resins were 73.1 and 238 °C for G and HT resins, respectively. Therefore, at high temperature, the HT resin was expected to be much more performant than the standard G resin. Typically, UV curing increases the tensile and flexural strength (UTS and FSB, respectively) and the stiffness (Young’s modulus E), but it decreases the elongation at break A%, therefore also increasing the brittleness. The effects of post-processing are summarized in Table 3.

The layer thickness was set to 25 μm in order to achieve the highest resolution and part accuracy. The slicer software Formlabs Preform (release v.3.3.3) was used to preprocess the part geometry (STL file) and generate supports and laser paths on layers (Figure 4c) by properly choosing the algorithm parameters. The size of the support attachment points was set to 0.6 mm; the support density was set to a medium level (1 into the range 0.5–1.5); the thickness of the base platform, also referred to as the raft, was set to 2 mm; and the minimum distance of the part from the raft was set at 5 mm. Part orientation, defined by the spatial placement of the object within the build volume of the machine, affects part stability, surfaces finishing, printability, and total processing time. Here, the mold inserts were placed with the back (planar face opposite the cavity) on the x–y plane and then rotated 20 degrees around the *x*-axis as shown in Figure 4c. This parameter was also chosen considering the anisotropy of the printed part due to the slicing, support positions, and part features such as holes, cavities, etc. 

**Figure 5:**
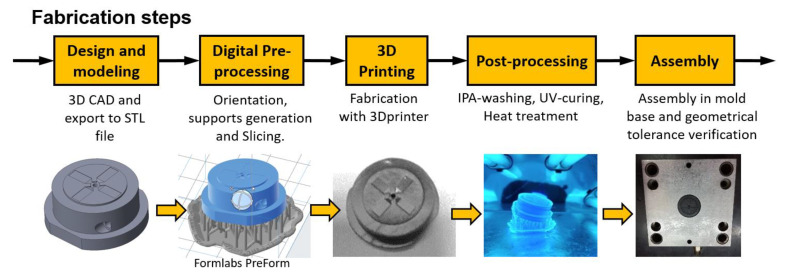
Mold insert fabrication steps.

The 3D-printed samples were washed in a Formlabs Form Wash machine for 20 min in high-purity 99.9% isopropyl alcohol (IPA) in order to remove the liquid resin on the part surfaces. All supports were removed with a cutter and finally the samples were post-processed with UV exposure performed in a Formlabs Form Cure machine and heat treatment (only HT resin after UV curing) in laboratory oven (UNE400, Memmert, Schwabach, Germany) (Table 4) [29,30]. In this application, each mold insert required about 9 milliliters of material but different processing times: 4 h for the G resin and 8.5 h for the HT resin. After the 3D printing, washing and UV curing took about 2.5 h and annealing (only HT resin) required 3 h. The fabrication and assembly steps of the AM mold insert are represented in Figure 5. 

The bottom-up (inverted) SLA technology is affected by three main accuracy issues: minimum size planar feature (laser spot diameter), minimum layer thickness (*z*-axis resolution), and geometrical part distortion due to the peeling mechanism of the cured layer. For these reasons, the assembly of the SLA mold insert into the steel mold requires a final adjustment on mating surfaces by means of manual tools and finishing abrasive paper. In order to guarantee a satisfactory cavity filling and avoid flashings, the parallelism of matching surfaces should be verified and deviation should be limited below a tolerance target. Injection molding clamping can completely or partially compensate for a low deviation depending on the clearance fit between the mold base and the SLA mold insert. 

## 4. Mold Insert Metrological Characterization

**Figure 6:**
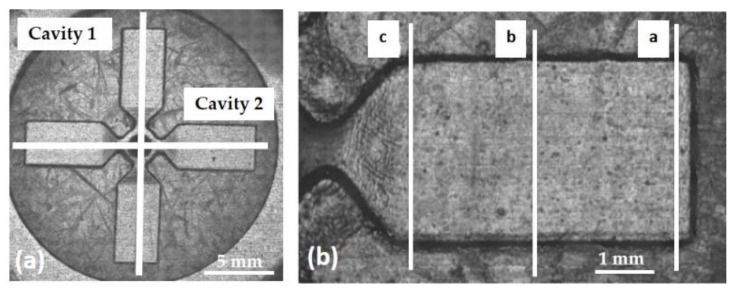
(**a**) Z-scan image of the mold insert; cross lines trace the directions for planarity measurements. (**b**) Z-scan image of cavity 1; the three lines trace the scanning direction adopted for depth measurements.

The SLA mold inserts were dimensionally characterized by a confocal microscope (CSM 700, ZEISS, Milan, Italy) in order to measure the real depth of the manufactured cavities. In addition, the planarity of the insert parting surface was evaluated once the mold was assembled. For both inserts, two of the four cavities were selected (1 and 2 in Figure 6a) and a topographic Z-scan acquisition was carried out by adopting a vertical resolution of 0.2 µm. Figure 6b shows the positions where depth measurements were performed at distance of 5.0 mm (a), 2.6 mm (b), and 0.2 mm (c) from the gate. For both cavities, the average values of the three measurements performed on each line are reported in Table 5 with the corresponding standard deviations and deviations from the nominal value of D_N_ = 100 μm. The deviation Δ*μ_i_*% is defined by Equation (1).

(1) $$\Delta \mu_{i}\%=\frac{\mu_{i}-D_{N}}{D_{N}}\times 100$$

From Table 5, the deviation Δ*μ*% assumed values between –7.4% and +9.4%, thus giving an experimental assessment of the dimensional accuracy of SLA in fabricating the mold inserts. 

For the G resin insert, the resulting values were very close to nominal ones (100 µm), as verified by a double t-sample test. A small difference was observed along line (c), and this was mainly due to the position near the gate having a higher depth according to the design specifications. For the HT insert, the depth measurements between the two cavities were slightly different but acceptable. These results confirm the effectiveness of SLA technology in the fabrication of mold inserts with thin cavities with accurate dimensions. 

Table 6 reports the diameter of the central hole measured for both inserts. In particular, the HT insert was measured before and after the last thermal treatment. It is possible to notice that the G insert presented a diameter very close to the nominal value, whereas the hole diameter of the HT insert was slightly bigger and was barely influenced by the thermal annealing. The difference was probably due to the combination of the SLA process and the HT material during fabrication.

Finally, the planarity of the G insert was verified by measuring the differences in the height between the upper parting surface of the SLA insert and the grinded steel plate of the mold once assembled. The measurements were performed along the bisection line of the cavities (crossed lines in Figure 6a) by Z-profile acquisitions. Along the radial direction on cavity 1 a difference of 50.0 ± 3.6 µm was measured, whereas a negligible difference (1 ± 0.9 µm) was measured along the cavity 2 direction. These findings show that the mold insert parting surface had a small slope angle of about 0.16°, referred to as the grinded mold surface. This deviation was caused by the peeling mechanism in the SLA fabrication, which, considering the part orientation in the build volume, affects the planarity tolerance of the surface. An unlevelled insert could cause poor mating between parting surfaces during mold closure, maybe compromising, partially or totally, the injection process due to polymer leakage. Therefore, as stated above, the mold insert assembly is a critical operation that requires excellent accuracy for the assessment and adjustments.

## 5. Micro-Injection Molding 

Injection molding is one of the most widely used forming operation and also one of the most complex, since there is a large number of process variables and flow conditions. Furthermore, producing micro components is an even more challenging task, requiring sizes with utmost precision. The reliable manufacturing of polymer-based miniaturized components is directly connected to the capability of controlling the micro injection molding procedure and is usually associated with the ability to completely fill the micro-size cavities of the mold during processing. The optimization of the process for a given product requires the adjustment of several parameters, especially injection velocity, mold and melt temperatures, and pressure, each taking different values at different stages of the process. Quality responses are usually associated with the evaluation of the replication by complete filling of the mold cavity. The experimental setup is reported next.

**Figure 7:**
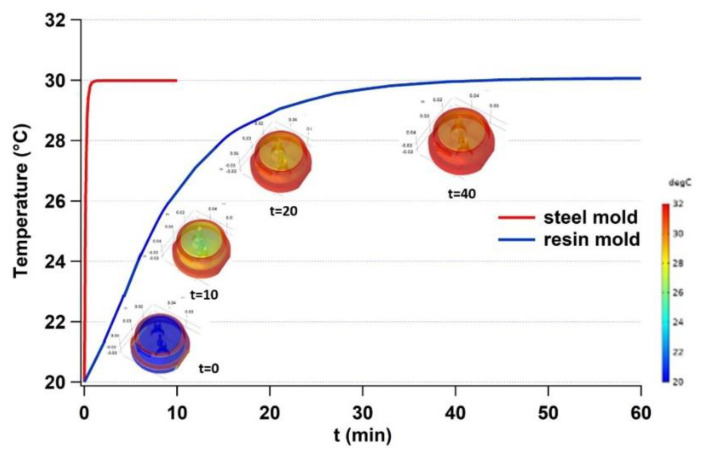
Time evolution of insert temperature and simulated image of resin insert after 0, 10, 20, and 40 min from initial heating.

A pre-heating time estimation of the mold was performed via simulation, since the temperature at the core of the insert measured experimentally could not be reliable due to poor heat transfer characteristics of the insert materials. The mold, formed by plates and insert, was modeled in FE software COMSOL Multiphysics^®^ release v5.5 to simulate the heat transfer, using the material properties corresponding to both SLA resins retrieved from software’s library data sheets. In particular, the thermal conductivity assigned in the model was equal to 0.19 W/mK. In the model, heat transfer was due to the contact faces of the heated plate and mold insert, whereas dissipation was due to the air convection of the external faces, assuming a transmission coefficient of 10 W/mK. The starting temperature of the mold plate was set to 30 °C. Figure 7 shows the increase in the temperature of the insert over time. The stationary condition at 30 °C was reached on the resin mold insert after about 30 min, whereas the same temperature was reached on the steel mold after 30 s. This time was therefore selected for waiting before starting the experimental trials. 

Since the part is very thin and injection side of the mold is made of steel, a difference in thermal behavior between the steel and polymeric inserts was not expected. Further simulations confirmed this statement.

A semi-crystalline thermoplastic polymer, polyoxymethilene (POM) (Ultraform, Basf, Ludwigshafen, Germany) was chosen for this study. The adopted POM belongs to the family of engineering thermoplastics. It has a partially crystalline structure with a high degree of crystallization, depending on process parameters, and it presents an ideal combination of strength, stiffness, and toughness. This combination of characteristics, in association with good tribological properties, makes it very suitable for molding applications. Table 7 reports the relevant POM properties. The material was dried before using for 4 h at temperature of 80 °C, as suggested by the manufacturer. 

The insert, realized via SLA, was mounted on the mold base and then assembled in a micro-molding machine (FormicaPlast 1K, DesmaTec, Achim, Germany). It is characterized by a two-piston architecture: one for material pre-plasticizing (diameter 6 mm) and the second for injection (diameter 3 mm) [31]. The motion is provided by a servo-electric driven unit capable of a maximum injection speed of 500 mm/s and a maximum injection pressure of 3000 bar. The piston has a maximum acceleration of 6 m/s^2^ and injection volume of 150 mm^3^. During the process, the material, in form of granules, is transferred from a hopper into a plasticizing unit so that it becomes molten and soft. The material is then forced under pressure into a mold cavity, where it is subjected to holding pressure for a specific time to compensate for material shrinkage. After a sufficient time, the material freezes into the mold cavity, gets ejected, and the cycle is repeated.

Several inserts in G resin were printed in order to screen suitable process parameters until the most favorable set (Table 8) was identified by visual inspection of the samples. Three values for injection speed were used in order to evaluate the infill capability of these challenging micro-features, whereas an injection speed of 130 mm/s was set to assess insert life. The mold temperature was kept at 30 °C to avoid tool degradation of the G resin mold insert. In addition, a low setting of holding pressure and injection speed could be used to prevent premature failure of the insert. In this case, this consideration was applied for pressure (100 bar) but was not possible for velocity (150 mm/s). In fact, due to the thin geometry of the features, a high injection speed was crucial to fill the cavity and prevent freezing.

After the time necessary to reach the thermal steady state, a short series of 30 samples was molded, evaluating both the capacity to fill the thin cavities and the capability of the SLA mold insert. 

## 6. Results and Discussion

An important aspect to be considered is mold wear due to the melt flow and cavity filling that occurs at high temperatures, generally around the polymer’s melting point or higher. In this experiment, the injection speed was set at 130 mm/s and the melt temperature was kept at 230 °C, as is typical for POM. It must be considered that the melt temperature is particularly critical for SLA G resin since its glass transition temperature (Tg) is about 75 °C [16]; thus, during molding the insert could be heated above the Tg.

**Figure 8:**
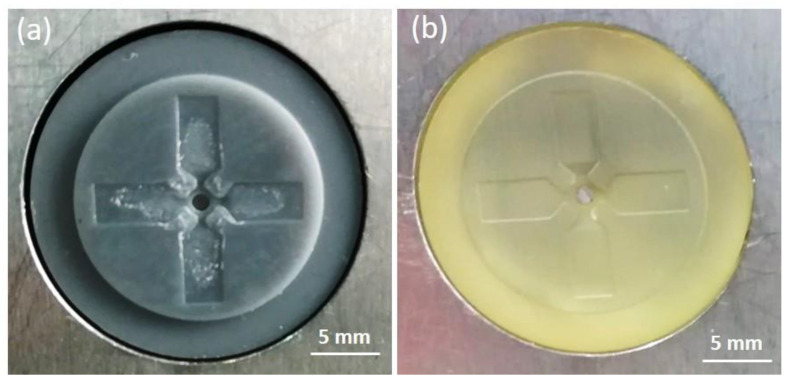
Insert pictures after 30 cycles: (**a**) chipping damage locations on the G resin insert and (**b**) undamaged HT insert.

The G resin insert life was evaluated by the number of shots and, after the 30 cycles, a chipping damage occurred in the insert near the gates where a higher melt pressure was experienced (Figure 8a). On contrary, considering the same operative condition, no damages occurred on the HT resin mold insert (Figure 8b). 

**Figure 9:**
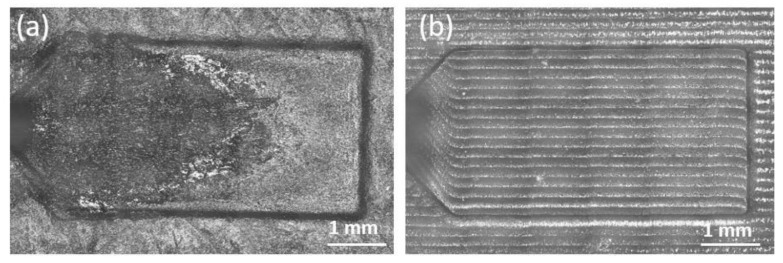
Confocal image of cavity 1 after 30 cycles: (**a**) grey resin insert and (**b**) HT insert.

Along the flow path, high temperature gradients and local melting of the skin of the mold and material removal could appear. This wearing mechanism can significantly compromise the life of the mold since it results in accuracy loss and geometry out-of-tolerance, as shown in Figure 9a for the G insert. The damage visible on the G insert had the typical parabolic shape of the melt front, rounded far from the gate due to the temperature reduction. In addition, the HT resin insert was tested in the same process condition, revealing no wear after 30 molding cycles (Figure 9b). After use, the two molds were measured again in the same previous conditions and the results are shown in Table 9. The depth variations after 30 μ-IM cycles Δ*μ_i_*_,30_% are defined by Equation (2).

(2) $$\Delta \mu_{i,30}\;\%=\frac{\mu_{i,0}-\mu_{i,30}}{\mu_{i,0}}\times 100$$

The measured depths and calculated variation show that the cavity wear occurred in the G resin insert, whereas HT resin insert did not undergo significant variation. In particular, the increment percentage of the G insert in cavity 2 was about +21% in correspondence to all measurement sections (a, b, c; see Figure 6), whereas cavity 1 reported a damage of +21.4% only at section c, very close to the gate were the melt front had the higher pressure and temperature values.

**Figure 10:**
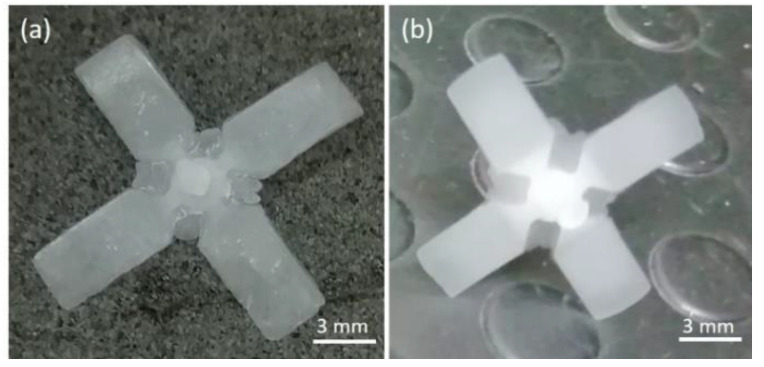
Injected parts: (**a**) by mean of grey resin insert and (**b**) by HT resin insert.

The obtained specimens presented (i) flashes in the gate area, probably due to the insert damage and high pressure during processing, and (ii) incomplete filling of the final part because of the melt freezing (Figure 10). Through simulation, it was also evaluated that a flattening of 20 µm of the resin insert occurred due to the applied pressure. Ten samples, produced with both inserts, were randomly collected and classified using confocal microscopy Zeiss CSM 700 by measuring the length of the flow front and the thickness of two perpendicular wings obtained in cavity 1 and 2 (Figure 6a). The mean length of samples, obtained by the grey resin insert, was 4.96 ± 0.48 mm in cavity 1 and 4.29 ± 0.34 mm in cavity 2. Since the nominal length was 5 mm, the replicability in the two cavities reached a mean value of 92.5%. The measured sample thickness was 174.8 ± 2 µm in cavity 1 and 169 ± 1.9 µm in cavity 2. These values were higher than the nominal cavity one probably due to the planar gap of the grey insert with respect to the half-mold surface that was not corrected properly in assembly and due to the mold wear increasing during molding. The mean length of the samples obtained by the HT resin insert was 4.90 ± 0.04 mm in cavity 1 and 4.89 ± 0.07 mm in cavity 2, showing a replicability of almost 98%. The samples thickness was 117.3 ± 0.9 μm in cavity 1 and 115.1 ± 0.9 μm in cavity 2. The results obtained with the HT insert were quite better than the grey one. 

**Figure 11:**
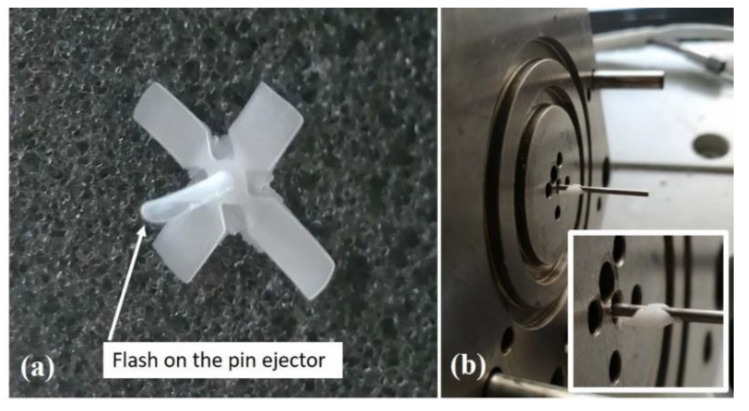
HT insert failure after 55 injection molding cycles: (**a**) flash on molded part and (**b**) burr on pin ejector.

After the first part of the experimentation of the manufacture of 30 samples, the HT insert was mounted again in the mold and an additional injection molding campaign was performed in order to investigate its failure mechanisms and lifetime. The same parameters reported in Table 9 were set for this additional campaign in order to keep the same experimental conditions as the previous one. After an additional 25 cycles, the molded parts started to exhibit flash at the tip of the pin ejector, evidencing the wear of the HT insert due to the sliding of the pin ejector during part extraction. The failure is reported in Figure 11 and this type of failure was never reported in previous studies. Of course, continuing with additional molding cycles resulted in the worsening of the flash extension until the extraction function of the mold was completely lost and the process was stopped.

**Figure 12:**
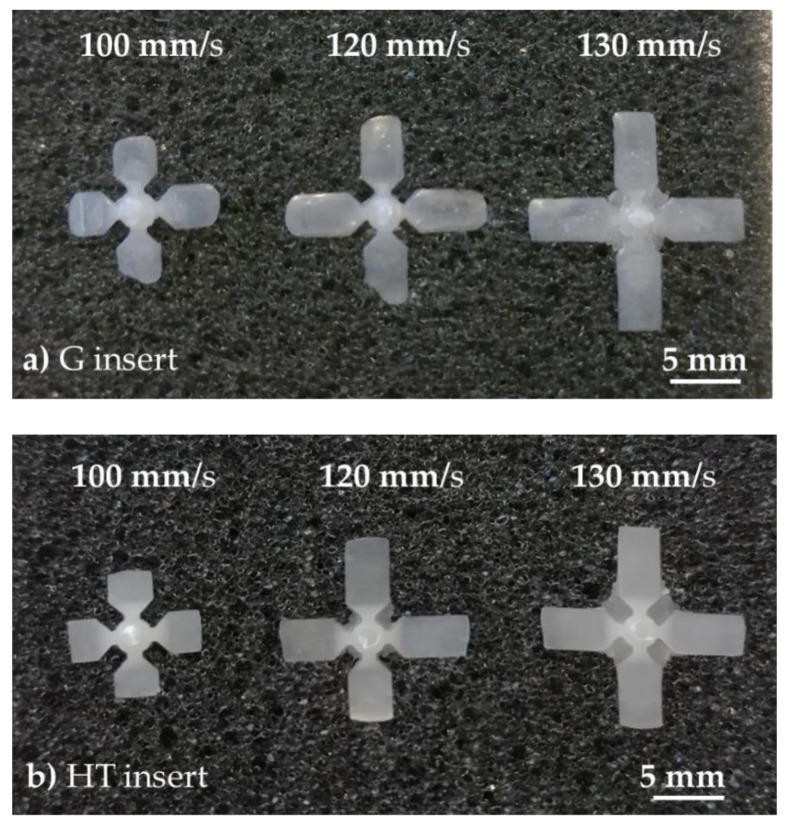
Infill variation at different injection speeds: (**a**) G insert and (**b**) HT insert.

A further investigation was finally performed considering three levels for the injection speed (100, 120, and 130 mm/s) in order to evaluate the cavity filling, avoiding the flash formation around the gate. The filling lengths are reported in Table 10, where it is possible to observe that the G insert had better results. However, it must be underlined that the G insert presented wear damage that increased the depth of the cavities at its beginning, helping the filling of the melt. This statement is confirmed by the specimen thickness and indirectly by Figure 12. 

As illustrated in Figure 12, the increasing of the injection speed produced not only a growth of the filling length but also flashes in the center of the specimens, that is, the zone of the insert subjected to the highest melt temperature and pressure. In particular, for the G insert, the flash appeared at the injection speed value of 130 mm/s, whereas for the HT insert flashes it started earlier, at injection speed of 120 mm/s. 

## 7. Conclusions

In this work, a thin cavity mold was manufactured via stereolithography and tested via injection molding in order to assess the feasibility of the SLA/µ-IM process chain. Design rules for additive manufacturing were applied in order to prevent injection molding issues and ensure a longer mold insert endurance. Two kinds of inserts were produced by SLA with different resins and were measured, showing good dimensional and thickness accuracy, with deviations from nominal values below 9%. The assembly of the insert into the base mold was instead affected by parallelism deviation due to accuracy in mechanical couplings and to the mold insert geometric tolerances. These deviations were mainly attributed to the mold insert distortion induced by the SLA peeling mechanism, as in the case of the measured planarity deviation of about 50 μm on the parting surface for the grey insert. However, the assembly issue was almost resolved using the HT resin insert. 

A numerical simulation was performed to assess the thermal behavior of the SLA resin insert when it replaces a steel one. The performed analysis suggested that, before molding, it is necessary to warm up the polymeric insert for 30 min.

Finally, a pre-series of about 30 molding cycles was performed and, although the molded thin features were affected by some defects, the replication accuracy was quite good regarding the cavity filling. In particular, the cavity filling was investigated while considering two possible results: maximum infill accepting flashes only in the center of the insert, and maximum infill without flashes. For the first goal, the samples obtained with the grey insert presented a replicability of 92%, whereas for the HT insert it reached 98%. For the second goal, using the grey insert the samples presented an infill of 4.26 ± 0.34 mm with an injection speed of 120 mm/s, whereas with the HT insert the samples infill was reduced to 2.05 ± 0.11 mm with an injection speed of 100 mm/s. However, the apparently better performance of the G insert was probably due to the damages that increased the cavity depth, helping the filling.

Regarding the mold damage, the grey insert showed wear due to the high-temperature polymer injection in the cavities, with increments of cavity depth up to 21%. This differed for the HT insert, which was still perfect after 30 molding cycles with no relevant damages measured. This material removal along the flow path was due to high temperature gradients and local melting of the skin of the mold. After 55 cycles, the HT mold insert exhibited flashes on the pin ejector caused by the wearing of the pin sliding in the molded part extraction. This failure, never reported in previous studies, is quite severe since it definitively causes the loss of the function of the molded part extraction.

This research showed that micro-injection molding process using an SLA mold insert are feasible and affordable but require accuracy, especially in soft-insert fabrication, assembly, and process parameter selection to prolong the tool life. AM and, in particular, SLA, should not be conceived as a replacement for injection molding or other traditional technologies for polymers, but they do have the potential to improve them, ensuring complex and accurate molds for small batches in a quick and low-cost way.

## Figures and Tables

**Table 1:** Performance of AM technologies for the fabrication of mold inserts with micro-features for μ-IM.

Performance	High-Precision AM Technology		
	SLA	Polyjet	DLP
Micro-features	Cantilever 4 × 0.5 mm thickness: 30–120 μm	Ribs	Bricks 5 × 4 mm and cylindrical pillars ∅200 × H200 µm
Planar XY resolution	56 μm	200 μm	50 μm
Layer resolution (thickness)	30 μm	16 μm	50 μm
Surface finishing	-	R_t_ = 6–11 μm	-
Mold insert endurance (μ-IM shots)	5	116	110
Failure mechanisms	Cracking and chipping, wear of cavity	Cracking and wear of cavity	Cracking and chipping, wear of cavity
Materials	VisiJet FTX Green photopolymer	Objet Digital ABS photopolymer	Methacrylic photopolymer resin
References	[24]	[21]	[15,16,17,18,19,20,22,23]

**Table 2:** Mechanical and thermal properties of Formlabs resins [28].

Material Properties			Formlabs Photopolymer Resins				
			Standard Grey G Code FLGPGR04		High Temperature V02 HT Code FLHTAM02		
Mechanical and thermal property	Units	ASTM Test Method	Green	UV Cured ^1^	Green	UV Cured ^1^	UV Cured + Heat Treatment ^2^
Density	gr/cm^3^	-	1.16	1.17	-	1.19	-
Ultimate tensile strength (UTS)	MPa	D638-14 D638-10	38	65	20.9	58.3	48.7
Elongation at break A%	%		12	6	14	3.3	2.3
Tensile modulus E	GPa		1.6	2.8	0.75	2.8	2.8
Flexural strength at break (FSB)	MPa	D790-15 D790-10			24.1	94.5	2.8
Flexural modulus	GPa		1.3	2.2	0.69	2.62	2.8
Impact, notched IZOD	J/m	D256-10	16	25	32.8	18.2	16.9
Coefficient of thermal expansion (0–150 °C)	μm/m °C	E831-13			118.1	79.6	74.5
Heat deflection Temperature (HDT) @0.45 MPa	°C	D648-16 D648-07	49.7	73.1	49	120	238
Heat deflection temperature (HDT) @1.8 MPa	°C	D648-16 D648-07	42.7	58.4	44	78	101

^1^ Exposure to 1.25 mW/cm^2^ of 405 nm LED light for 60 min at 60 °C. ^2^ UV curing for 120 min at 80 °C; heat treatment for 180 min at 160 °C.

**Table 3:** Effects of post-processing (UV curing and heat treatments) on SLA resins.

Material Properties	Units	UV Curing		UV + Heat Treatment
		G	HT	HT
		Δ%	Δ%	Δ%
Ultimate tensile strength (UTS)	(MPa)	+71	+179	+133
Elongation at break A%	(%)	–50	–76	–84
Tensile modulus E	(GPa)	+75	+273	+273
Flexural strength at break FSB	(MPa)	-	-	–88
Flexural modulus	(GPa)	+69	+280	+306
Impact, notched IZOD	(J/m)	+56	–45	–48
Coefficient of thermal expansion (0–150 °C)	(μm/m °C)	-	-33	–37
Heat deflection temperature @0.45 MPa	(°C)	+47	+145	+386
Heat deflection temperature @1.8 MPa	(°C)	+37	+77	+130

**Table 4:** Post-processing cycles (UV and heat treatments) applied to materials.

Post-Processing	SLA Resin	
	Standard Grey V04	High Temperature V02
UV curing ^1^	60 min @ 60 °C	120 min @ 80 °C
Heat treatment	-	180 min @ 160 °C

^1^ Exposure to 39W UV light, wavelength λ = 405 nm.

**Table 5:** Cavity depth of mold inserts and deviations from nominal value.

	G Insert				HT Insert			
	Cavity 1		Cavity 2		Cavity 1		Cavity 2	
	μ ± SD	Δ*μ*%	μ ± SD	Δ*μ*%	μ ± SD	Δ*μ*%	μ ± SD	Δ*μ*%
Line (a)	97.1 ± 0.9	–2.9	96.9 ± 1.3	–3.1	102.1 ± 1.9	+2.1	93.9 ± 3.4	–6.1
Line (b)	97.1 ± 1.9	–2.9	101.2 ± 1.6	+1.2	106.2 ± 2.6	+6.2	92.6 ±. 2.6	–7.4
Line (c)	109.4 ± 1.8	+9.4	105.7 ± 3.1	+5.7	104.1 ± 2.5	+4.1	95.3 ± 1.9	–4.7

**Table 6:** Central hole diameter of the inserts.

Insert	Post-Processing	Diameter (mm)	SD (mm)
Grey	curing	1.316	0.009
HT	curing	1.389	0.016
	curing + annealing	1.398	0.013

**Table 7:** POM properties.

Name	Trade Name	Grade	Manufacturer	MVR (cm^3^/10min) (ISO 1133)	Density (kg/m^3^) (ISO 1133)	Tensile Modulus (MPa) (ISO 527)
POM	Ultraform	N2320 003	Basf	7.5	1400	2700

**Table 8:** Micro-injection molding process parameter settings.

Process Parameter	Value
Melt temperature	230 °C
Mold temperature	30 °C
Injection speed	100, 120, 130 mm/s
Holding pressure	100 bar
Holding time	1 s
Cooling time	5 s
Piston stroke	3.5 mm

**Table 9:** Cavity depth of inserts after molding.

	Depth After 30 Shots (µm)							
	G Insert				HT Insert			
	Cavity 1		Cavity 2		Cavity 1		Cavity 2	
	μ ± SD	Δ*μ*%	μ ± SD	Δ*μ*%	μ ± SD	Δ*μ*%	μ ± SD	Δ*μ*%
Line (a)	94.1 ± 0.5	-3.1	117.9 ± 6.0	+21.7	103.2 ± 1.3	+1.1	94.8 ± 2.4	+1.0
Line (b)	100.6 ± 1	+3.6	122.2 ± 8.0	+20.8	104.9 ± 1.8	-1.2	96.6 ± 0.9	+3.2
Line (c)	132.8 ± 2.6	+21.4	127.1 ± 4.4	+20.2	102.7 ± 1.8	-1.3	100.9 ± 3.4	+5.9

**Table 10:** Filling length at different injection speeds and insert materials.

Insert Resin	Injection Speed (mm/s)	Average Filling Length (mm)	Standard Deviation (mm)
Grey	100	2.68	0.37
	120	4.26	0.30
	130	4.99	0.09
HT	100	2.05	0.11
	120	4.16	0.57
	130	4.87	0.30
